# Noninvasive Glucose Monitoring with a Contact Lens and Smartphone

**DOI:** 10.3390/s18103208

**Published:** 2018-09-22

**Authors:** You-Rong Lin, Chin-Chi Hung, Hsien-Yi Chiu, Po-Han Chang, Bor-Ran Li, Sheng-Jen Cheng, Jia-Wei Yang, Shien-Fong Lin, Guan-Yu Chen

**Affiliations:** 1 Institute of Biomedical Engineering, College of Electrical and Computer Engineering, National Chiao Tung University, Hsinchu 300, Taiwan; yoronglin@gmail.com (Y.-R.L.); jhincihong19901008hy@gmail.com (C.-C.H.); liborran@gmail.com (B.-R.L.); shengjen@nctu.edu.tw (S.-J.C.); jiawei@nctu.edu.tw (J.-W.Y.); linsf5402@nctu.edu.tw (S.-F.L.); 2 Department of Electrical and Computer Engineering, College of Electrical and Computer Engineering, National Chiao Tung University, Hsinchu 30010, Taiwan; 3 Department of Dermatology, National Taiwan University Hospital Hsin-Chu Branch, Hsinchu 30059, Taiwan; extra.owl0430@yahoo.com.tw; 4 Institute of Biomedical Engineering, College of Medicine and College of Engineering, National Taiwan University, Taipei 10051, Taiwan; 5 Department of Dermatology, National Taiwan University Hospital, Taipei 10002, Taiwan; 6 Department of Dermatology, College of Medicine, National Taiwan University, Taipei 10051, Taiwan; 7 Department of Applied Chemistry, National Chiao Tung University, Hsinchu 30010, Taiwan; mattc34011239@gmail.com; 8 Department of Biological Science and Technology, National Chiao Tung University, Hsinchu 30010, Taiwan

**Keywords:** noninvasive, glucose monitoring, contact lens, smartphone, image detection

## Abstract

Diabetes has become a chronic metabolic disorder, and the growing diabetes population makes medical care more important. We investigated using a portable and noninvasive contact lens as an ideal sensor for diabetes patients whose tear fluid contains glucose. The key feature is the reversible covalent interaction between boronic acid and glucose, which can provide a noninvasive glucose sensor for diabetes patients. We present a phenylboronic acid (PBA)-based HEMA contact lens that exhibits a reversible swelling/shrinking effect to change its thickness. The difference in thickness can be detected in a picture taken with a smartphone and analyzed using software. Our novel technique offers the following capabilities: (i) non-enzymatic and continuous glucose detection with the contact lens; (ii) no need for an embedded circuit and power source for the glucose sensor; and (iii) the use of a smartphone to detect the change in thickness of the contact lens with no need for additional photo-sensors. This technique is promising for a noninvasive measurement of the glucose level and simple implementation of glucose sensing with a smartphone.

## 1. Introduction

Diabetes is one of the most common metabolic disorders in the world, with more than 400 million people currently affected [1,2]. It is mainly characterized by a chronically raised glucose concentration in the blood. Uncontrolled diabetes may lead to serious damage to several organs, such as the heart, blood vessels, eyes, kidneys, and nerves [1,3]. One strategy for measuring the glucose level is enzyme-based chemical sensors. Among the various enzyme-based sensors [4], the most well-known is glucose oxidase (GOx) [5]. Immobilization of the GOx enzyme during electrochemical transduction can be used as a bio-recognition element. The GOx enzyme can be used with a conductive material as a sensing electrode to detect glucose based on the reduction/oxidation of the electrode by H_2_O_2_ [6,7]. Another sensor is based on protein: the competitive and complementary binding properties of synthetically glycosylated insulin and glucose to concanavalin A (Con A) [8]. However, enzyme- or protein-based compositions can be denatured or limited to environmental changes. It would affect the functionality of the system after it has been used and stored for extended periods of time [9]. A fast and reliable method that allows for the construction of non-enzyme smart polymers for glucose sensors would thus be useful.

Phenylboronic acids (PBAs) are Lewis acids that can bind reversibly to cis-1,2 or cis-1,3 diols, including glucose, to form a stable five-member ring complex. Extensive studies have investigated the binding affinity of boronic acids with different diols including fructose, glucose, and other sugars [10,11,12]. HEMA (poly(2-hydroxyethyl methacrylate)) hydrogel contains neutral boronic acid groups that are generally hydrophobic, whereas the anionic boronate groups become water-soluble. As the concentration of glucose increases, the ratio of the anionic forms to the neutral form becomes larger, which increases the hydrophilicity of the system. This manifests as reversible covalent bonding [13]; such a property can serve as an ideal candidate for sensor design [14].

Contact lenses, which people mainly wear for vision correction and cosmetic reasons, can now provide another unique function as a wearable device for the real-time monitoring of the glucose level of diabetes patients. Apart from the material construction of the contact lens, its potential as a point-of-care (POC) and portable device is also crucial for blood glucose monitoring. Important factors include portability, cost effectiveness, and easy use for rapid and accurate diagnosis to reduce time spent and costs [15,16,17,18]. Portable diagnostic tools include blood pressure sensors [19], cellphone-based rapid lateral flow test readers [20], cellphone microscopy [21,22,23], and handheld optical coherence tomography scanners [24]. Optical imaging and image processing techniques make a critical contribution to these POC devices [25,26]. Because there is a correlation between tear fluid and blood, tear glucose can serve as a surrogate for blood analysis. In terms of glucose dynamics, there is a 15 to 30 min time delay of the tear fluid level after glucose is transported from the blood level [27,28]. Monitoring the glucose level is the widely used diagnostic standard for determining the best treatment for diabetics [29]. Noninvasive detection of the glucose level has been carried out in numerous ways to replace conventional invasive diagnostic tests. Invasive techniques for blood glucose monitoring, such as pricking the finger for a blood sample, would be painful for patients [30,31,32]. The contact lens can serve as a portable noninvasive blood glucose measurement device for continuous health monitoring when equipped with a sensor to detect physiological changes and metabolites in tears [29,33,34,35]. Although such a system provides many capabilities, some issues remain, including the encapsulation of electronic chip materials and metal wireless circuits for the sensor in the contact lens. A concern might be that such components may affect the user’s vision [33,36]. Implanted integrated circuit (IC) chips, rigid interconnects, semiconductor electronics, and the power source may damage the cornea or eyelid [37]. A different technique described the approach of optical near infrared spectroscopy and new glucose responsive materials based on polymer crystalline colloidal arrays or boronic acid containing fluorophores to detect glucose in tears through the color change. However, in their research, they lack an excitation and detection device. Furthermore, some people may not get used to the color of a doped lens [38,39,40].

**Figure 1:**
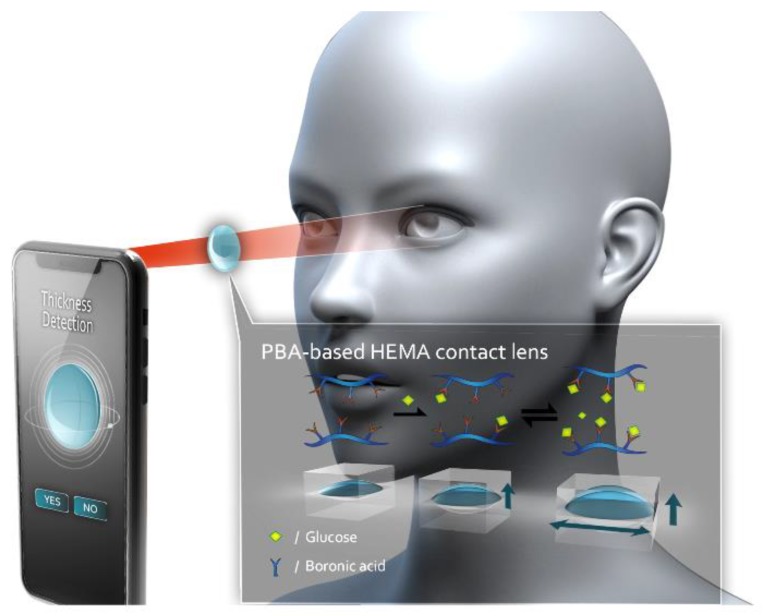
System of using a smartphone for detecting the thickness of the PBA-based HEMA contact lens. A portable noninvasive contact lens with an imaging program in a smartphone for an ideal method for sensing diabetes patients whose tear fluid contains glucose. The key feature is the reversible covalent interaction of boronic acid in HEMA and glucose. The volume of the PBA-based HEMA changes simultaneously with the glucose level.

Here, we propose a portable and optical device combined with an imaging process: the non-enzyme Phenylboronic acid (PBA) based Hydroxyethyl methacrylate (HEMA) contact lens, PBA-based HEMA contact lens (Figure 1). When the PBA-based contact lens absorbs glucose, it swells; this increases the thickness of the contact lens. We utilized this property to develop a reversible hydrogel that generates differential osmotic pressure for continuous sensing. The PBA-based HEMA contact lens can function as a sensitive glucose sensor that changes thickness with different glucose concentrations. After our light-emitting diode (LED) device emits light, the change in thickness can be detected using a smartphone. Our PBA-based contact lens may be a notable alternative to invasive diagnostic monitoring such as pricking the finger for diabetic patients.

## 2. Materials and Methods

### 2.1. Fabrication of the Polydimethylsiloxane (PDMS) Samples

To obtain polydimethylsiloxane (PDMS) samples with different thicknesses, the weights of Sylgard-184A and Sylgard-184B (Dow Corning, Auburn, MI, USA) were set to a ratio of 10:1. We then placed the mixture into a vacuum to remove air bubbles. The three different weights (150, 900 and 2000 mg) of mixture were then poured onto the mold, and were well-distributed using the rotator shaker (30 rpm) for 10 min at room temperature. After curing at 80 °C for 24 h, the samples were demolded and the thickness was measured using a micrometer screw gauge (Mitutoyo, Japan).

### 2.2. Fabrication of the PBA-Based HEMA Contact Lens

The fabrication of the PBA-based HEMA contact lens followed three steps: (i) synthesis of HEMA-OTs; (ii) fabrication of the contact lenses; and (iii) modification of 3-phenylboronic acid on HEMA-PBA. First, to obtain HEMA-OTs, 1 g (7.68 mM) of 2-hydroxyethyl methacrylate was dissolved in anhydrous dichloromethane. A fresh solution of 1.6 g (8.39 mM) of p-toluenesulfonyl chloride and 1.1 g (9 mM) of 4-(dimethylamino)-pyridine was prepared in 5 mL of anhydrous DCM and slowly added into the HEMA solution while being stirred. This was then stirred for 8 h at room temperature (RT). To purify the reaction solution, 10 mL of a 0.5 N HCl water solution was added for washing. This was followed by washing with 10 mL of saturated NaHCO_3_. The DCM layer was dried with anhydrous Na_2_SO_4_ and concentrated under reduced pressure.

Second, the poly-HEMA contact lens was prepared as follows. To avoid the double bond portion of 2-hydroxyethyl methacrylate, which is easily attacked by the amine group of 3-phenylboronic acid, HEMA and HEMA-OTs were decided prior to polymerization. The double bond portion of 2-hydroxyethyl methacrylate, which is easily attacked by the amine group of 3-phenylboronic acid, HEMA and HEMA-OTs were decided prior to polymerization. HEMA and HEMA-OTs were well mixed at a ratio of 9:1 for a total amount of 500 µL. Then, 2.5 µL of ethylene glycol dimethacrylate and 2.5 µL of Darocur 1173 were added to the solution while being stirred. After being stirred for 10 min in a dark environment, 35 µL of the mixture solution was added to each contact lens mold. Solidification proceeded under 365 nm UV light for 20 min. The contact lenses were demolded after the whole mold had been soaked in water for 8 h. 

Finally, 3-phenylboronic acid was modified on the fabricated poly-HEMA contact lens as follows. First, 10 mg of 3-phenylboronic acid was dissolved in 2 mL ethyl acetate, and 7.4 mg of trimethylamine was added to the solution. After that, HEMA-PBA was washed using ethyl acetate for 1 h, soaked in the 3-phenylboronic acid solution and reacted at 40 °C overnight. HEMA-PBA was then washed with MeOH and stored in deionized (DI) water. The final product was a PBA-based HEMA contact lens.

### 2.3. Characteristics of the PDMS Samples and PBA-Based HEMA Contact Lens

Two types of material were utilized in this study: the PDMS samples and a PBA-based HEMA contact lens. PDMS was the prototype for the thickness test. Different PDMS thicknesses were prepared (0.135, 0.973, 2.247, and 5.343 mm). PBA-based HEMA was the final material of the contact lens, and all samples were fabricated following the above process. 

### 2.4. Glucose Response of the PBA-Based HEMA Contact Lens

We prepared different concentrations of the glucose solvent to observe the change in thickness of the PBA-based HEMA. d(+)-glucose(glucose monohydrate) (Sigma, Neustadt an der Weinstraeße, Germany) was used to prepare a standard glucose solution containing phosphate buffered saline (PBS) (2.7 mM of KCl and 138 mM of NaCl) buffered at pH 7.2 ± 0.2 at RT. During the experiment, the dish was filled with 1.5 mL of 0.1 and 0.6 mM glucose solution at pH 7.2 ± 0.2 for 6–8 h. Then, a digital microscope (Leica DVM6, Japan) was the tool used to measure the thickness of the PBA-based HEMA contact lens sample. We cut the contact lens from the edge spread out flat and observed the cross section (thickness).

### 2.5. Light-Emitting Diode (LED) Detector Module for Detecting Thickness

A detector device with red light LED emissions was used and the images taken using the smartphone camera. We utilized this platform to detect changes in the red light to reflect the contact lens thickness. The red LED light-emitting diode (LS-F3URC-YBS) with a wavelength of 620–630 nm was mounted on a circuit module as the excitation light source and powered by an external direct current (DC) power supply (MSP-100-15 MEAN WELL) [41]. Then, the Sony IMX230 photosensitive sensor already embedded in the smartphone was used to detect the red light. The data measured using the smartphone were analyzed with MATLAB.

### 2.6. Real-Time Image Processing

The changes in the glucose level were induced using different concentrations of glucose solution and monitored with pictures taken using a smartphone (HTC M9+ with a Sony IMX230 photosensitive sensor). In order to perform an initial light area detection over each picture, the smartphone camera was switched to manual mode with the same settings and conditions, such as the RAW file format, a shoot speed of 1/125, and an ISO value of 200. After the picture was taken, we utilized MATLAB as a platform to analyze the image data. The red light contact lens image was changed from the RGB color space to gray levels to segment the area of interest. After that, the appropriate threshold was set to find the outer circle of interest. Erosion was applied to remove small objects from the binary image to decrease the environmental noise. To obtain the ratio of the outer circle to the red area, we enhanced the red color domain to find the red area. During this process, we found the endpoints of the circle and segmentation in the space domain in order to target the red-light area and reference. The red area was divided by the outer circle; the ratio can be considered as the change in red light emission and thus thickness.

### 2.7. Continuous Glucose Monitoring 

This was carried out to confirm the feasibility of continuous glucose monitoring using the red light-emitting device and smartphone. Here, we prepared two different glucose concentrations of 0 mM and 20 mM, respectively. The samples were immersed in 1.5 mL of glucose solutions with different levels of concentration, and each sample was separately soaked for 2 h to complete a dynamic reaction within 10 h. Finally, this process was repeated for two cycles to continue recording the differences between two levels of glucose concentration according to our red light-emitting device, smartphone and real-time image processing.

### 2.8. Cell Cytotoxicity Analysis

An in vitro test was performed to examine the cell cytotoxicity of the co-incubated PBA-based contact lens. ARPE-19 cells (a human retinal pigment epithelial cell line) were routinely cultured and passaged in a cell culture dish at 37 °C and 5% CO_2_ using a DMEM medium supplemented with 10% Ham’s F12 medium (Gibco, Thermo Fisher). The cells were harvested after 24 h and then incubated with individual glucose contact lenses for an additional 8 h. After being co-incubated for 8 h, the cells were stained with trypan blue and quantified as percentages of viable cells with the Cellometer^®^ Auto T4 Plus cell counter (Nexcelom Bioscience, Lawrence, MA, USA).

### 2.9. Statistical Analysis

Data were represented as the mean ± standard deviation based on at least three independent capture experiments. 

## 3. Results and Discussion

**Figure 2:**
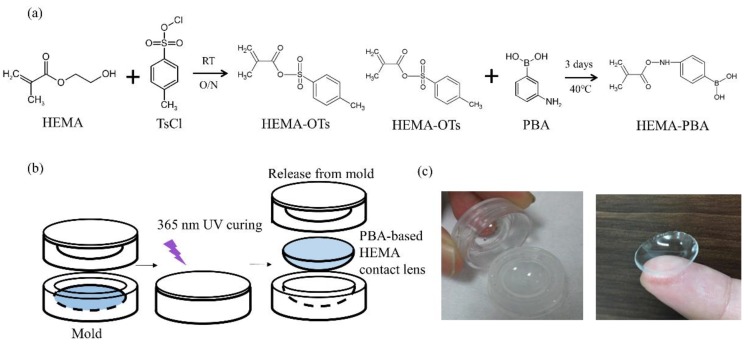
Synthetic process and fabrication of the PBA-based HEMA contact lens. (**a**) To obtain a contact lens sensitive to the glucose level. The synthesis process of the PBA-based HEMA contact lens including HEMA-OTs, fabrication of the contact lenses, and modification of 3-phenylboronic acid on HEMA-PBA. (**b**) Schematic of the PBA-based contact lens molding process. HEMA-PBA mixture was added to each mold, and cured with 365 nm UV. Then, the PBA-based HEMA contact lenses were formed in the mold, then demolded. (**c**) The photograph of the PBA-based HEMA contact lens was fabricated using (**b**).

To obtain a contact lens sensitive to the glucose level, we synthesized a PBA-based contact lens by introducing the property of boronic acids into an amphiphilic polymer gel structure (HEMA). PBA-based HEMA exhibits reversible volume change behavior (volume phase transition) driven by the change in the osmotic pressure synchronized with the change in glucose concentration. The assembly process and final product of the PBA-based contact lens are shown in Figure 2a,b. Figure 2c shows our successfully fabricated soft contact lens with high transmissibility and stretchability.

**Figure 3:**
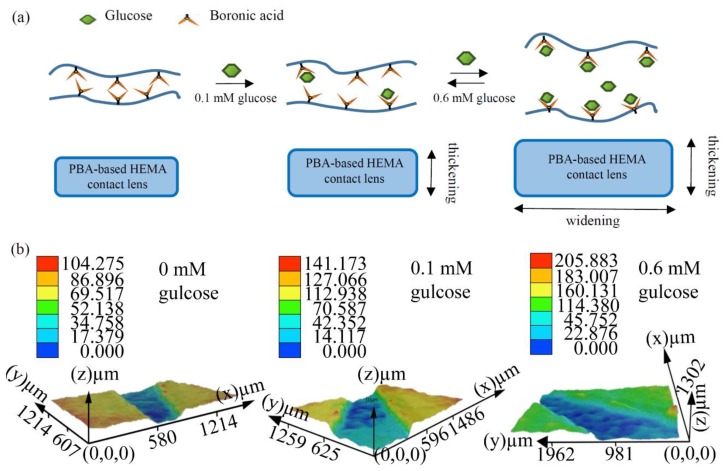
Properties of the PBA-based HEMA contact lens at different glucose levels. (**a**) Schematic illustration of the PBA-based HEMA contact lens. Principle of glucose detection in different glucose concentrations (0, 0.1 and 0.6 mM). (**b**) PBA-based HEMA contact lens at different glucose levels as observed with a Leica DVM6 digital microscope. Different colors represent the distance in the *z*-axis between the underside and surface of the contact lens.

Next, we introduced this contact lens glucose sensor to different glucose levels to observe the swelling/shrinking effect. The glucose level in the tears of normal people ranges from 0.1 to 0.6 mM [42]. People diagnosed as diabetic patients have a tear glucose level higher than 0.61 mM [43]. Figure 3a,b shows images taken by the Leica DVM6 digital microscope. The thickness of the PBA-based contact lens was constant in the phosphate buffered saline (PBS) solution and served as a control group for comparison with other groups with a glucose solution (0.1 and 0.6 mM). To observe the thickness of the PBA-based contact lens, we cut the sample from the edge and then spread the contact lens out flat on the slides. The data showed that the cross section of the PBA-modified contact lens at 0.6 mM was about 91–137 µm. The thickness of the PBA-based contact lens at 0.1 mM ranged from 98 to 112 µm, and at 0 mM ranged from 70 to 86 µm (Figure 3b). The volume of the contact lens increased with the glucose concentration. Overall, these results show that the volume change of the contact lens has great potential for glucose sensing.

**Figure 4:**
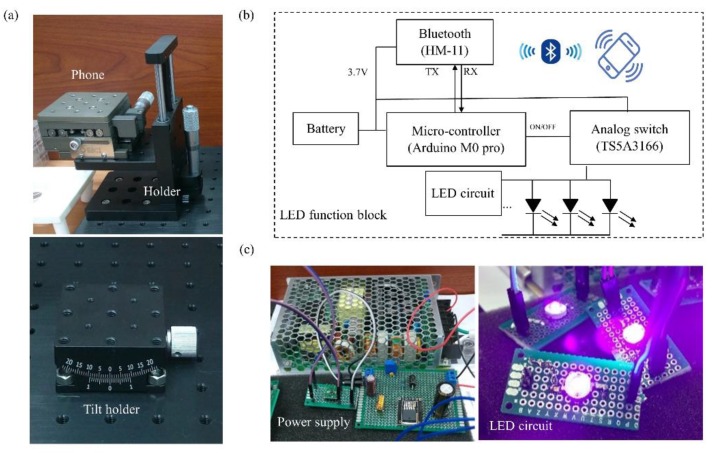
System hardware design for detecting the thickness of a PBA-based HEMA contact lens. (**a**) A stable distance for detecting the thickness of the PBA-based HEMA contact lens was used as the platform with a smartphone (HTC M9+ with a Sony IMX230 photosensitive sensor). (**b**) The schematic illustration of the system hardware configuration of the emission device. (**c**) Photograph of a microcontroller (Arduino M0 Pro, left) powered by a 3.7 V power supply and Bluetooth module (HM-11, right) that could be controlled using a smartphone-triggered LED light and receive the image easily.

The smartphone was used as a device for the noninvasive detection of the changes in thickness of the PBA-based contact lens. In the first prototype (Figure S1), we used a 3D printer to print our holder and red-light emission device. Our holder material was polylactic acid (PLA). The power supply device supplied electric power of 3.7 V to the LED circuit. Figure 4a and Figure S2 present the static platform used to place the smartphone to regularize the distances of the sample and contact lens. As the image resolution and background noise were the two main concerns for our device, we set up the platform to overcome these obstacles. Figure 4b shows the function block of the red LED light device. A microcontroller (Arduino M0 Pro) powered by a 3.7 V power supply and a chip (Texa Instruments, LM2596) were included in the Printed Circuit Board(PCB) layout circuit to convert 110 V power to DC voltage supply (3.7 V). The Bluetooth module (HM-11) that could be controlled by the user’s smartphone triggered the LED light and received the image easily (Figure 4c). The platform provided a fixed distance in the experiment. We used the CMOS light detect sensor already embedded in the smartphone to detect the red light. In other words, this application can be easily applied and merged with other applications on a smartphone. Compared to existing contact lens sensors [25,33,44], our sensor is novel because it does not contain circuits or an enzyme and is combined with a smartphone. This wearable contact lens can not only provide real-time detection of the glucose level without obstructing vision but also allow for easy image detection with a smartphone without the need for any other complex and expensive sensors to detect the thickness.

**Figure 5:**
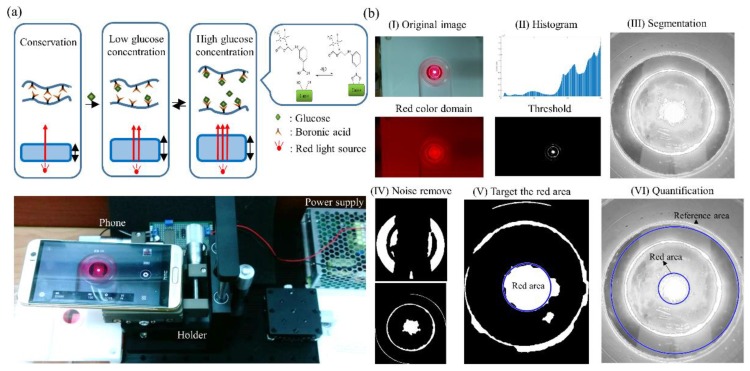
Automatic recognition of the thickness of the PBA-based HEMA contact lens glucose sensing using imaging processing. (**a**) Schematic of the PBA-based HEMA contact lens at different glucose levels. Different colors represent differences in the z-axis between the underside and surface of the contact lens (Top). Photograph of the platform with a smartphone used as the red-light source detection device (Down). (**b**) Photograph of the imaging processing: (I) Original image is segmented into three color domains, RGB converted to the gray level and the threshold to a binary image; (II) perform pre-segmentation on the area of interest and set the threshold; (III) find endpoints in the binary image and perform segmentation in the space domain for a morphologically close image; (IV) remove small objects from the binary image; (V) target the red light area and reference; (VI) measure the properties of the imaged regions and quantify the ratio of the red light area and reference.

**Figure 6:**
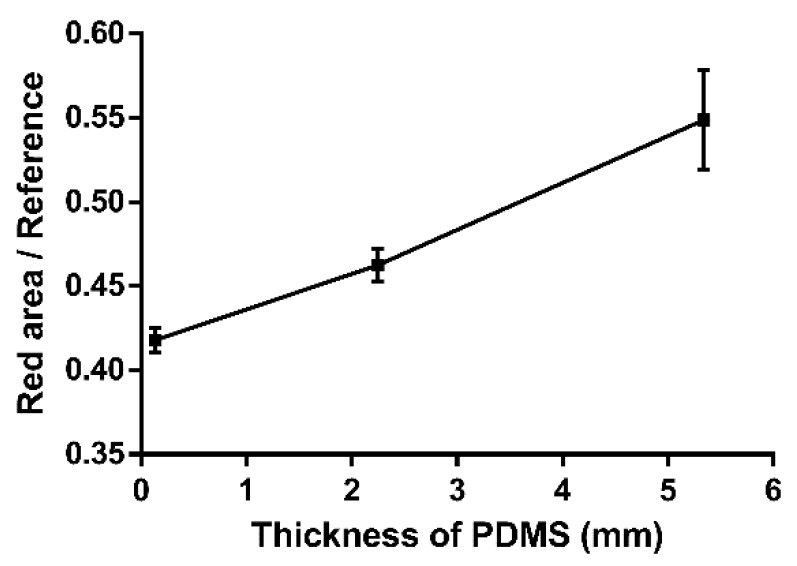
Calibration curve for the image detection ratio and thickness. The parameters are calculated according to the area of the red-light circle divided by the area of the sample edge. Three different thicknesses were considered: 0.135, 2.247 and 5.343 mm. The parameters were 0.418, 0.462 and 0.549, respectively.

Figure 5a provides the working principle and experiment in glucose sensing. We then observed the difference of red-light emission, while the optical power changed with the thickness of the PBA-based contact lens. Optical power is the degree to which a lens converges or diverges light. Since the contact lens becomes thicker, Gullstrand’s equation can be applied to thick lenses to calculate the relation between optical power and the thickness of the contact lens [45,46,47]. After that, the change in value was detected after an image was taken using the smartphone. Data were loaded into the software for analysis, and the image processing program could automatically recognize the edge of the contact lens and compute the thickness difference. During this process, the background noise was filtered so that we could focus on the area of interest in the PDMS sample. Figure 5b shows the picture after it was transformed into a binary image. Then, a multi-level threshold method [48,49] was applied to remove the scatter of the red light source and unwanted elements. We computed the background value of the pixels and the value of the targeted red area. Thus, we found our area of interest to enhance the features [50]. Taking these particular features into consideration, we could find the center of the area and reconstruct a circle. Based on the obtained results, this circle was labeled as the “red area,” and the edge of the sample was labeled as the “red reference” (see Figure 5b, V–VI). The parameters could be computed from the area of the red-light circle divided by the area of the sample edge. As the sample thickness and red-light emission were correlated following the change in surface powers, different parameter values could be obtained. The parameter values corresponded to each sample thickness. Figure 6 shows the quantification results for the red area/reference of circular PDMS samples with several different thicknesses. The reference area was represented as the whole circle edge of the PDMS sample. The circular PDMS sample (radius 2 cm) image was detected, and the area of interest was segmented [51]. We also had to confirm the capability of this method of sensing tear fluids in a real environment. The main factor of our concern was the relation between the thickness and the parameter value. Three different thicknesses were prepared: 0.135, 2.247, and 5.343 mm (Figure S3). The resulting parameter values were 0.418 ± 0.007, 0.462 ± 0.010, and 0.549 ± 0.030, respectively. Thus, our platform could detect different thicknesses of the PDMS samples.

To verify that the PBA-based contact lens is nontoxic and harmless to the human body, the cell viability of ARPE-19 was measured using the Cellometer^®^ Auto T4 Plus cell counter (Nexcelom Bioscience, Manchester, UK). Figure S4 shows that both the control group and the PBA-based HEMA had no significant cell death. The in vitro test clearly showed that our PBA-based contact lens has high biocompatibility. Finally, we demonstrated that, in addition to noninvasive detection of the glucose level, this technique offers repeatability and continuous sensing.

**Figure 7:**
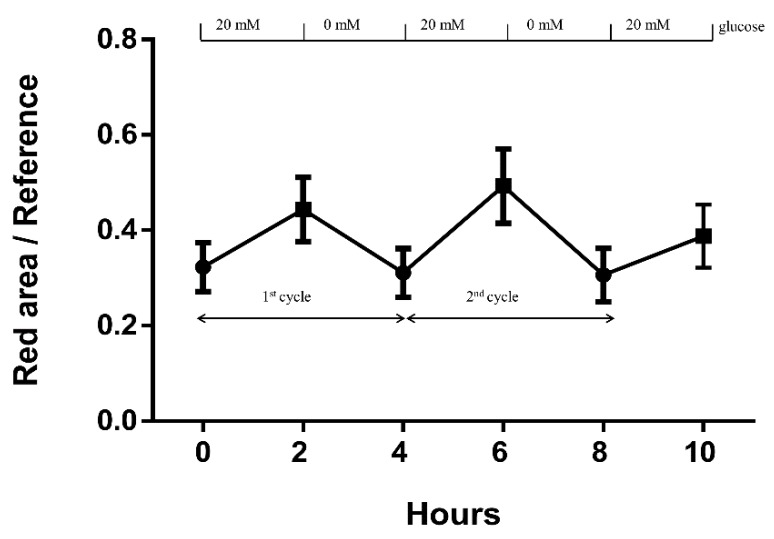
Continuous and reversible glucose sensing. PBA-based HEMA contact lens for continuous sensing in 0 and 20 mM glucose solvent. The trial was repeated three times.

Figure 6 shows the image response for continuous sensing at a glucose level of 20 mM. Figure 6a shows the experimental setup for glucose sensing. The contact lens was put on the platform, and the image was taken using an HTC M9+. In the experiment, our PBA-based HEMA gel allowed about a 90% response time when exposed to glucose solution within 15 min. Figure 7 shows that the PBA-based HEMA contact lens could continuously detect different concentrations of the glucose solvent: 0 and 20 mM. The contact lens was soaked in 0 mM for 2 h; thereafter, it was soaked in 20 mM glucose solvent and recorded using a smart phone. The PBA-based contact lens absorbed glucose and the parameter (red area/red reference) was raised. After 2 h absorption, it was returned to 0 mM glucose solvent. This process was repeated to confirm the repeatability of this glucose sensor. The experiment revealed completely reversible swelling/shrinking ability in three cycles to 20 mM glucose. In the MATLAB program, the red reference is the edge of the PBA-based HEMA contact lens, and the red area is the emission of red light. Those two areas can be detected automatically. The data plotted in Figure 7 highlight that our technique has greater potential for future applications involving continuous and noninvasive glucose monitoring for patients with diabetes.

## 4. Conclusions

We constructed non-enzyme smart polymers endowed with purely synthetic boronic acid (PBA) with a high affinity for glucose, i.e., PBA-based HEMA. This causes reversible deformation due to its swelling and shrinking behavior. The ability of boronic acid to reversibly form a complex with sugars has led to it being heavily employed as a glucose sensor. Moreover, our PBA-based HEMA contact lens combined with an image device can function as a glucose sensor. It provides several advantages. First, the contact lens is not modified with any additional enzyme. Second, the material offers reversible glucose sensing. Compared to existing contact lens sensors made of conventional opaque material or embedded with circuits [42,52], our flexible contact lens does not obstruct the patient’s vision. Third, the device is portable (merged with a smartphone). Therefore, the detection program can be easily implemented and enhanced without an additional reader. This new approach shortens the development cycle and offers the potential for point-of-care and intelligent healthcare.

## Supplementary Materials

The following are available online at http://www.mdpi.com/1424-8220/18/10/3208/s1, Figure S1. Prototype: Device of the detection and emission, Figure S2. Final device of the detection and emission, Figure S3. Illustration of detection process before and after the image processing steps and Figure S4. The cytotoxicity of PBA-based pHEMA contact lens against ARPE19.
