# Supplementary material for: Noninvasive Glucose Monitoring with a Contact Lens and Smartphone
Source: Sensors (Basel). 2018 Sep 22;18(10):3208. doi: 10.3390/s18103208 (PMC6210255; doi:10.3390/s18103208)
Supplement: Supplementary file 1 [file sensors-18-03208-s001.pdf]

Supplementary Materials

Noninvasive Glucose Monitoring with a Contact Lens and Smartphone

You-Rong Lin ,Chin-Chi Hung , Hsien-Yi Chiu, Bo-Han Chang , Bor-Ran Li , Sheng-Jen Cheng , Jia-Wei Yang , Shien-Fong Lin and Guan-Yu Chen

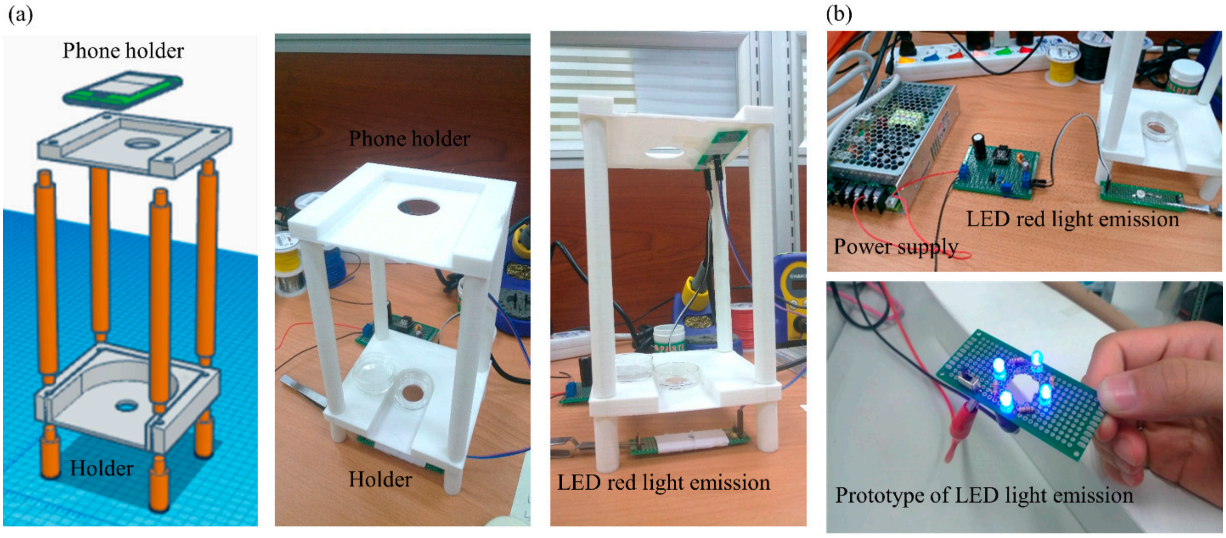

Figure S1. Prototype : Device of the detection and emission. (a) Photographs of device set up. In the first prototype, we use 3D printer to print our holder to provide a fixed distance in the experiment. Our material of holder is Poly Lactic Acid (PLA). (b)The power supply device supplies electric power 3.7V to the red light LED emission device.

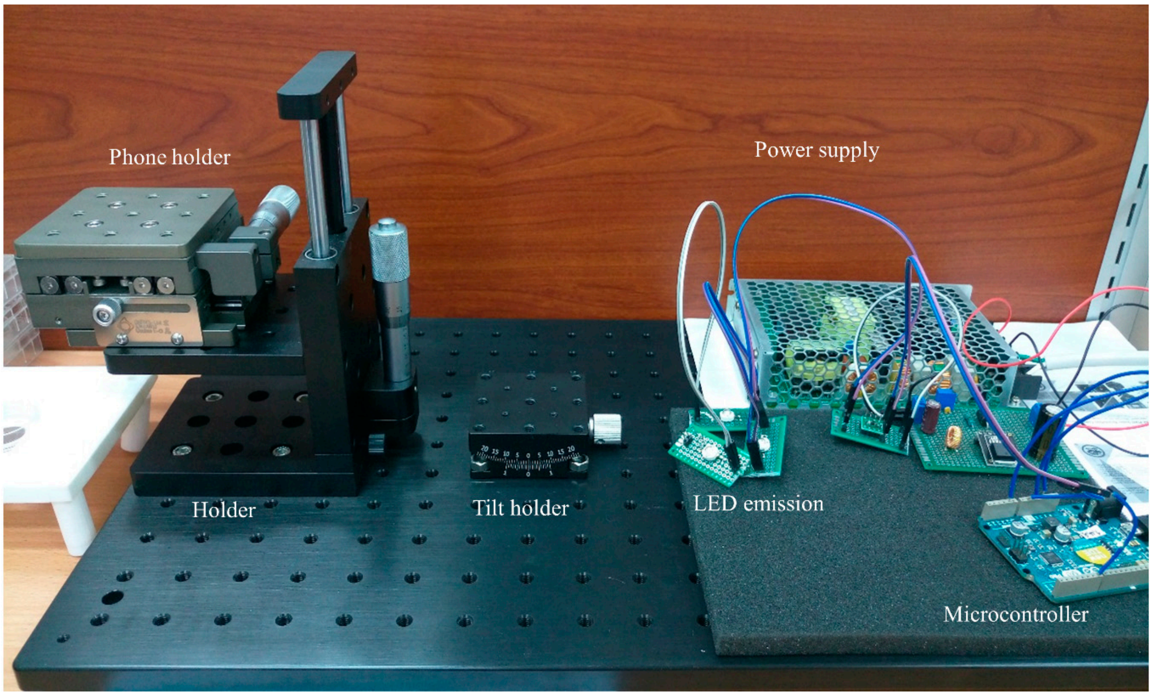

Figure S2. Final device of the detection and emission. We provide a statistic optical platform. A microcontroller (Arduino M0 Pro) and bluetooth module (HM-11) that can be controlled through smartphone for user can trigger LED light and receive the image easily. The platform provided a fix the distance in the experiment.

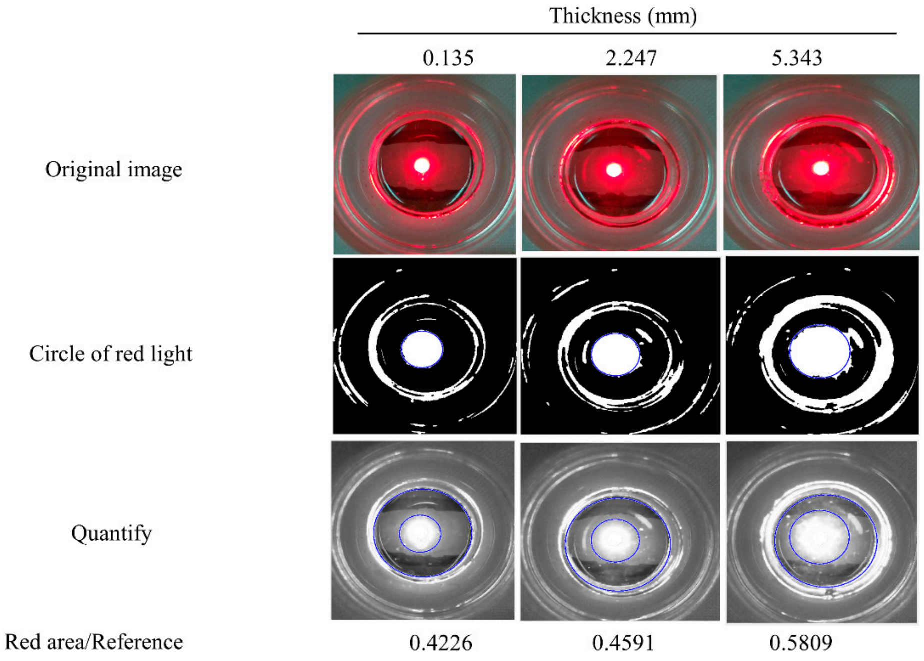

Figure S3. Illustration of detection process before and after the image processing steps. Three different thickness in this table: 0.135 mm, 2.247 mm and 5.343 mm. Picture was taken by HTC M9+ with a Sony IMX230 photosensitive sensor. All pictures were taken at the same setting and environment.

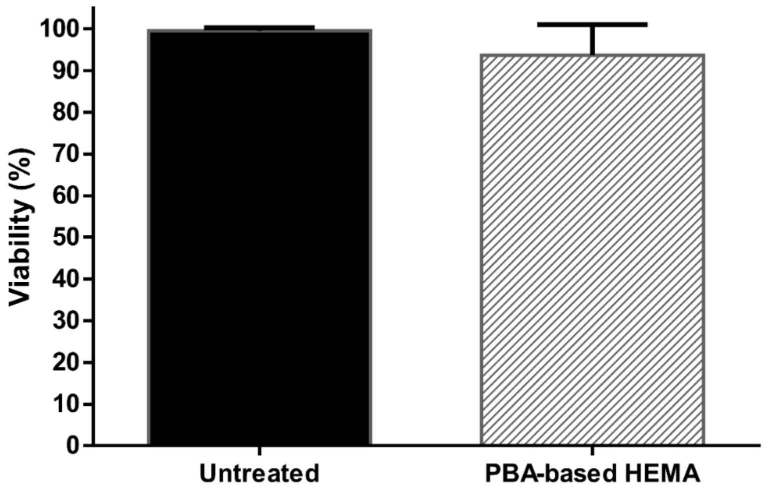

Figure S4. The cytotoxicity of PBA-based pHEMA contact lens against ARPE19. ARPE19 were seeded to 24-well plates ($5x10^4$ cells/well) and cultured overnight, washed with PBS and co-cultured with PBA based HEMA contact lens for 8 h in DMEM medium containing 10% FBS. Quantification of cell viability by automated cell counter. Quantification of cell viability by automated cell counter. Quantitative data represent the mean ± S.D. of at least 3 independent culture experiments.
